# The complete chloroplast genome of *Melicope pteleifolia* (Champ. ex Benth.) T. G. Hartley (Rutaceae)

**DOI:** 10.1080/23802359.2021.1909434

**Published:** 2021-04-05

**Authors:** Zhengjun Wu, Liwei Wu, Jianyong Xing, Yonghua Li, Yu Wang, Hui Yao

**Affiliations:** a China Resources Sanjiu Medical & Pharmaceutical Co., Ltd, Shenzhen, China; b Institute of Medicinal Plant Development, Chinese Academy of Medical Sciences and Peking Union Medical College, Beijing, China; c Guangxi University of Chinese Medicine, Nanning, China

**Keywords:** *Melicope pteleifolia*, chloroplast genome, phylogenetic analysis, medicinal plant

## Abstract

*Melicope pteleifolia*, an important medicinal and horticultural plant, has antipyretic, anti-inflammatory, and analgesic effects. Here, the complete chloroplast genome of *M. pteleifolia* was sequenced and its phylogenetic relationship was investigated. The complete chloroplast genome of *M. pteleifolia* was 159,014 bp in size, including a pair of inverted repeat regions (IR, 27,640 bp), a large single copy region (LSC, 85,124 bp), and a small single copy region (SSC, 18,610 bp). The GC content of the chloroplast genome was 38.3%. A total of 133 genes were annotated, including 88 protein-coding genes, 8 rRNA genes, and 37 tRNA genes. Phylogenetic analysis revealed that *M. pteleifolia* clusters together with species of *Toddalia*, *Zanthoxylum*, *Tetradium*, *Phellodendron*, and *Casimiroa*.

*Melicope pteleifolia* (Champ. ex Benth.) T. G. Hartley 1993 (synonym *Euodia lepta* (Spreng.) Merr. 1935) is an understory tree or shrub in family Rutaceae distributed in Fujian, Guangdong, Guangxi, Hainan, Jiangxi, Taiwan, Yunnan, and Zhejiang provinces of China and Southeast Asia (The Editorial Committee of Flora of China 2008). Stems and young leafy branchlets of this species are the primary components of the Chinese Patent Medicines “Sanjiu Weitai” and “Sanjiu Ganmaoling” and its leaves are used to prepare a popular herbal ice tea (Liu et al. 2016). Previous studies have identified the bioactive essential oils, flavonoids, and alkaloids in *M. pteleifolia* that impart its antipyretic, anti-inflammatory, and analgesic effects (Liu et al. 2016; Mahadi et al. 2016). Recent studies reported that isolates from *M. pteleifolia* exhibited the strong enzymatic inhibition and showed moderate reductions in H1N1-induced cytopathic effects (Nguyen et al. 2016). For an important medicinal and horticultural plant (Liu et al. 2016; Mahadi et al. 2016), there is a dearth of basic phylogenetic and genomic data to better understand its unique chemical composition and important pharmacological properties (Nguyen et al. 2016; Mahadi et al. 2016; Xu et al. 2019). Here, the complete chloroplast genome of *M. pteleifolia* is reported which will be an invaluable resource for species determination and phylogenetic studies.

Fresh leaves of *M. pteleifolia* were collected from Pingyuan county, Guangdong province (N24°30′24″, E115°47′9″) in China. The voucher specimen was deposited in the Herbarium of Institute of Medicinal Plant Development (Herbarium Code: IMD; collector: Zhengjun Wu; voucher: Y20011). Total genomic DNA was extracted according to the operating manual of the DNeasy Plant Mini Kit (Qiagen, Hilden, Germany). DNA concentration and quality were assessed using Nanodrop 2000C (Thermo Fisher Scientific Co., Waltham, MA) spectrophotometry and 1% (w/v) agarose gel electrophoresis, respectively. Using the NEBNext Illumina library preparation kit (Beijing, China), libraries were prepared for PE150 sequencing on the Illumina Hiseq X. Chloroplast genome assembly and annotation were conducted as previously described (Cui et al. 2019), and gene content comparison was analyzed by CPGAVAS2 (Shi et al. 2019).

The chloroplast genome of *M. pteleifolia* is 159,014 bp in length (GenBank accession number: MW046256), including a large single copy region (LSC, 85,124 bp), a small single copy region (SSC, 18,610 bp), and a pair of inverted repeat regions (IR, 27,640 bp). Total GC content is 38.3%. The GC contents of SSC, LSC, and IR regions are 32.8%, 36.6%, and 42.8%, respectively. A total of 133 genes were annotated, including 88 protein-coding genes, 8 rRNA genes, and 37 tRNA genes. Nine protein-coding genes (*rpl2*, *rpl22*, *rpl23*, *ycf2*, *ycf15*, *ndhB*, *rps7*, *rps19*, and *rps12*), seven tRNAs (*trnI-CAU*, *trnL-CAA*, *trnV-GAC*, *trnI-GAU*, *trnA-UGC*, *trnR-ACG,* and *trnN-GUU*) and four rRNAs (*rrn16*, *rrn23*, *rrn4.5*, and *rrn5*) are located in the IR regions. In protein-coding genes, the AT contents in the first, second, and third codon positions are 53.9%, 61.7%, and 68.5%, respectively. Among the protein-coding genes, 11 genes contained introns, of which 2 genes (*clpP* and *ycf3*) contain two introns, while the remaining 9 genes (*ndhA*, *ndhB*, *petB*, *petD*, *rpl16*, *rpl2*, *rpoC1*, *rps12*, and *rps16*) contain only one intron. *rps12* gene is a trans-splicing gene with 5′ end in LSC region and 3′ end in IR region.

To confirm the phylogenetic relationship of *M. pteleifolia*, a total of 20 chloroplast genome sequences of Rutaceae species, including all published Chinese genera, were downloaded from GenBank. All the sequences were aligned by MAFFT (Katoh and Standley 2013) and a phylogeny was reconstructed with maximum likelihood (ML) using IQTREE (Nguyen et al. 2015) with a bootstrap of 1000 repetitions using *Dimocarpus longan* as the outgroup (Figure 1). The results indicate that *M. pteleifolia* clusters together with species of *Toddalia*, *Zanthoxylum*, *Tetradium*, *Phellodendron*, and *Casimiroa*; all bootstrap values are 100%.

**Figure 1.**
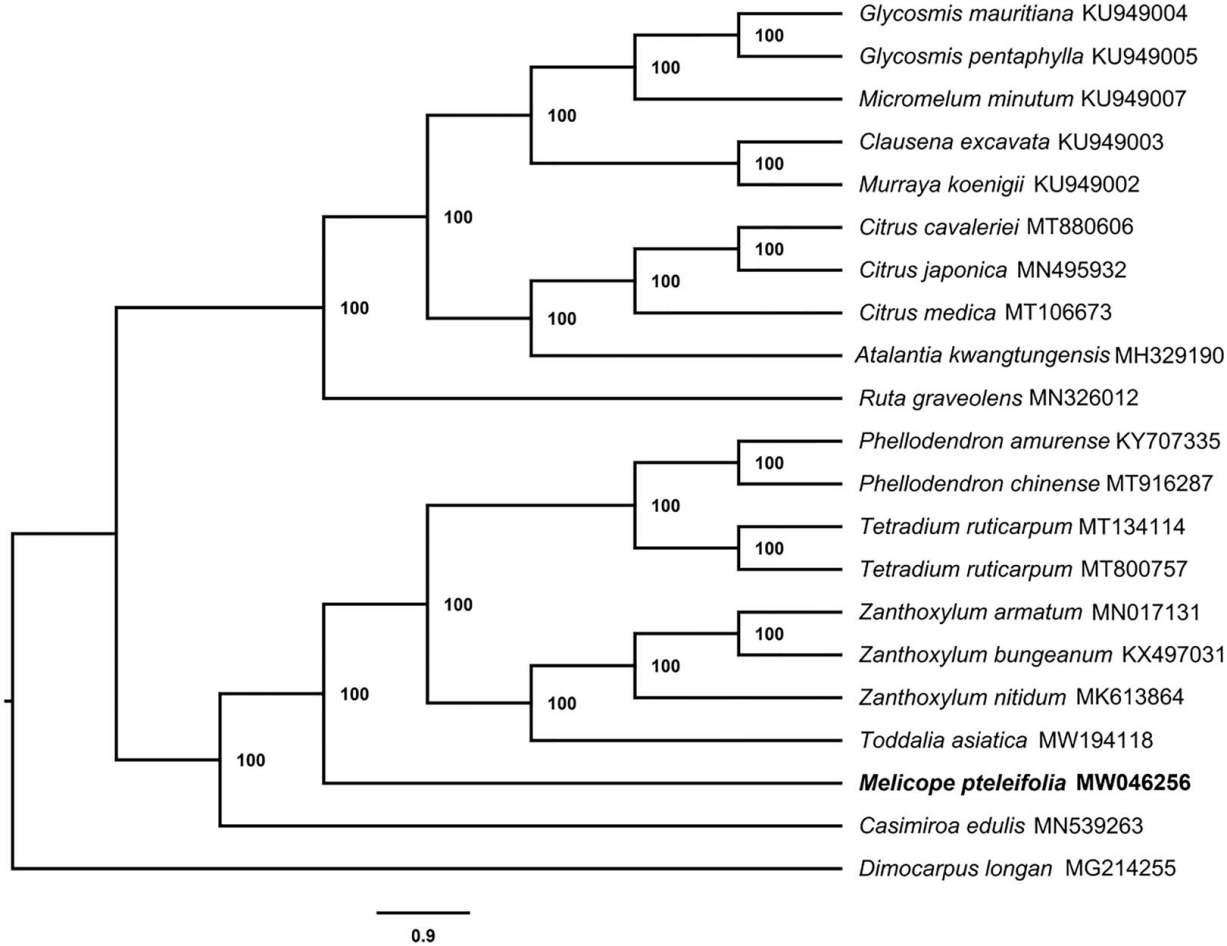
Phylogenetic tree of *Melicope pteleifolia* and other Rutaceae species using maximum-likelihood (ML) analyses based on complete chloroplast genome sequences. Bootstrap values are indicated on each node.

## Data Availability

The genome sequence data that support the findings of this study are openly available in GenBank of NCBI at https://www.ncbi.nlm.nih.gov/nuccore/MW046256 under the accession NO. MW046256. The associated BioProject, SRA, and Bio-Sample numbers are PRJNA689686, SRS7968375, and SAMN17214562, respectively.
